# Smad3 Inactivation and MiR-29b Upregulation Mediate the Effect of Carvedilol on Attenuating the Acute Myocardium Infarction-Induced Myocardial Fibrosis in Rat

**DOI:** 10.1371/journal.pone.0075557

**Published:** 2013-09-25

**Authors:** Jie-Ning Zhu, Ren Chen, Yong-Heng Fu, Qiu-Xiong Lin, Shuai Huang, Lin-Lin Guo, Meng-Zhen Zhang, Chun-Yu Deng, Xiao Zou, Shi-Long Zhong, Min Yang, Jian Zhuang, Xi-Yong Yu, Zhi-Xin Shan

**Affiliations:** 1 Medical Research Department of Guangdong General Hospital, Guangdong Provincial Cardiovascular Institute, Guangdong Academy of Medical Sciences, Guangzhou, China; 2 School of Pharmaceutical Sciences, Southern Medical University, Guangzhou, China; The Chinese University of Hong Kong, Hong Kong

## Abstract

Carvedilol, a nonselective β-adrenoreceptor antagonist, protects against myocardial injury induced by acute myocardium infarction (AMI). The mechanisms underlying the anti-fibrotic effects of carvedilol are unknown. Recent studies have revealed the critical role of microRNAs (miRNAs) in a variety of cardiovascular diseases. This study investigated whether miR-29b is involved in the cardioprotective effect of carvedilol against AMI-induced myocardial fibrosis. Male SD rats were randomized into several groups: the sham surgery control, left anterior descending (LAD) surgery-AMI model, AMI plus low-dose carvedilol treatment (1 mg/kg per day, CAR-L), AMI plus medium-dose carvedilol treatment (5 mg/kg per day, CAR-M) and AMI plus high-dose carvedilol treatment (10 mg/kg per day, CAR-H). Cardiac remodeling and impaired heart function were observed 4 weeks after LAD surgery treatment; the observed cardiac remodeling, decreased ejection fraction, and fractional shortening were rescued in the CAR-M and CAR-H groups. The upregulated expression of Col1a1, Col3a1, and α-SMA mRNA was significantly reduced in the CAR-M and CAR-H groups. Moreover, the downregulated miR-29b was elevated in the CAR-M and CAR-H groups. The *in vitro* study showed that Col1a1, Col3a1, and α-SMA were downregulated and miR-29b was upregulated by carvedilol in a dose-dependent manner in rat cardiac fibroblasts. Inhibition of ROS-induced Smad3 activation by carvedilol resulted in downregulation of Col1a1, Col3a1, and α-SMA and upregulation of miR-29b derived from the miR-29b-2 precursor. Enforced expression of miR-29b significantly suppressed Col1a1, Col3a1, and α-SMA expression. Taken together, we found that smad3 inactivation and miR-29b upregulation contributed to the cardioprotective activity of carvedilol against AMI-induced myocardial fibrosis.

## Introduction

Acute myocardial infarction (AMI) is one of the most serious cardiovascular conditions. The acute loss of myocardium post-AMI triggers a cascade of intracellular signaling processes, contributing to left ventricular (LV) remodeling, scar expansion, and heart failure. Beta-adrenergic receptor antagonists (β-blockers) effectively ameliorate post-AMI LV remodeling. Carvedilol, a third-generation, non-selective β-adrenoreceptor antagonist, possesses antioxidant, anti-apoptotic, anti-inflammatory, and anti-fibrotic properties [1]. Numerous studies have shown that carvedilol treatment improves the left ventricular ejection fraction (LVEF) and attenuates left ventricular remodeling in patients with chronic heart failure (CHF) [2]. The protective effects of carvedilol against AMI-induced myocardial injury have been attributed to the reduction of fibrosis [3,4]. However, the precise mechanisms underlying the anti-fibrotic effect of carvedilol is unknown.

MicroRNAs (miRNAs) are endogenous 20–23-nucleotide non-coding RNAs that negatively regulate gene expression at the posttranscriptional level by degrading or deadenylating target mRNA or by inhibiting translation [5]. MiRNAs have been reported to play key roles in diverse biological and pathological processes, including cell differentiation, proliferation, apoptosis, heart disease, neurological disorders, and human cancers [6-10]. Several miRNAs, including miR-21 [11], miR-1, miR-206 [12], miR-31, and miR-499-5p [13] are reported to be dysregulated in myocardial infarction, suggesting a fundamental role in AMI. MicroRNA-29b, a regulator of fibrosis [14-16], is dysregulated in AMI [14]. The TGF-β/Smad3-dependent pathway plays an important role in the pathogenesis of cardiac fibrotic and hypertrophic remodeling [17]. The effect of carvedilol on the inactivation of Smad3 signaling is unknown. In this study, we assessed the hypothesis that Smad3 signaling and miR-29b mediate the effect of carvedilol on attenuating AMI-induced myocardial fibrosis in rat.

## Materials and Methods

### Ethics Statement

Male sprague-dawley (SD) rats weighing 180-240 g, license number SCXK (YUE) 2004–0011 (Department of Experimental Animal Research Center, Sun Yat-sen Medical College, Sun Yat-sen University, Guangzhou, China) were used. All animals were housed on a 12-h light/dark cycle under pathogen-free conditions and kept on standard mouse chow with free access to tap water. This study conformed to the Guide for the Care and Use of Laboratory Animals published by the US National Institutes of Health (8th Edition, National Research Council, 2011). The present program was also approved by the research ethics committee of Guangdong General Hospital, the approval number was No. GDREC2010093A.

### A rat model of AMI and carvedilol treatment

AMI was induced by ligating the left anterior descending coronary artery as described [18]. Electrocardiography was used to demonstrate ST elevation and thereby confirm the success of surgery. Forty surviving rats that underwent ligation were randomly divided into five groups (N=8): (1) The Sham control group (Sham), (2) Saline treated AMI model group (AMI), (3) Low dose of carvedilol treated AMI group (CAR-L, 1 mg/kg/day), (4) Medium dose of carvedilol treated AMI group (CAR-M, 5 mg/kg/day), (5) High dose of carvedilol treated AMI group (CAR-H, 10 mg/kg/day). The second day after LAD surgery, four-week-oral dosing was conducted in rats in all groups except the sham group. All rats were fed ad libitum (the dose of standard rat fodder for one rat over 30 g/day).

### Echocardiographic study

Left ventricular (LV) function variables were assessed by transthoracic echocardiography. After the induction of light general anesthesia, the rats underwent transthoracic two-dimensional (2D) guided M-mode echocardiography with a 8.5-MHz transducer (Acuson, Mountain View, CA). From the cardiac short axis (papillary level), the LV anterior wall end-diastolic thickness (LVAWd), the systolic LV anterior wall thickness(LVAWs), the LV internal dimension at end-diastole (LVIDd), the LV internal dimension at end-systole(LVIDs), the LV posterior wall end-diastolic thickness (LVPWd), the LV posterior wall end-systolic thickness(LVPWs), the ejection fraction (EF) and fractional shortening (FS) were measured. Echocardiographic measurements were averaged from at least three separate cardiac cycles.

### Histological analysis

Rats were killed by an intraperitoneal injection of 2 mL of pentobarbital. The heart was excised, and the LV myocardium fixed overnight in 10% formalin. Samples were embedded in paraffin and cut into 4 µm thick sections. They were mounted on normal glass slides and stained with Masson trichrome for histological examination. For the collagen volume fraction (CVF) analysis in the border zone of the infarcted region, eight separate views (magnification=original×400) were selected and assessment of CVF used the following formula: CVF=collagen area/ total area.

### Rat cardiac fibroblasts (CFs) isolation, culture and treatment

CFs were isolated from 1-3 days old SD rats by using a modification of previous report [19]. CFs were separated from cardiomyocytes by gravity separation and grown to confluency on 10-cm cell culture dishes in growth media (DMEM/LG 10% FBS 1% penicillin 1% streptomycin) at 37 °C in humid air with 5% CO2. CFs from the third passage were used for experiments. The passage 3-4 neonatal SD rat CFs were used for mechanism study *in vitro*. Fifty nM Smad3 siRNA, 5 µM Smad3 inhibitor SIS-3, 80 µM Smad3 inhibitor naringenin (Nar) were used to treat rat CFs.

### Imaging of DCF Fluorescence

Intracellular oxidants in mouse cardiac fibroblasts were measured by using the probe dichloroﬂuoroscin diacetate (DCFH-DA) and confocal microscopy as described [20]. In brief, cells were incubated with DCFH-DA (10 µM) for 15 min, and subsequently treated with Hoechst33342 (1 µg/mL) for 15 min before being imaged. Cells were washed twice with culture medium, and images were acquired from five or more randomly chosen fields using Leica SP5 Spectral scanning laser confocal microscope (Leica Microsystems, Wetzlar, Germany). The levels of DCF fluorescence were analyzed with Leica Application Suite 2.02 software.

### Quantitative real-time PCR

Total RNA was extracted using TRIzol reagent (Gibco-BRL, Grand Island, NY, USA) according to manufacturer’s instruction. Methods for coding gene and miR-29b precursor expression detection were as follows: First-strand cDNAs were synthesized using a mixture of oligo (dT) 15 and random primers with Superscript reverse transcriptase (Invitrogen, Carlsbad, CA, USA). Real-time PCR was performed with the following thermal cycling profile: 95°C for 5 min, followed by 40 cycles of amplification (94°C for 25 s, 60°C for 25 s, and 72°C for 25 s). The absorption values of the SYBR Green I fluorescence in each tube were detected at the end of each cycle. The housekeeping gene GAPDH was used as an internal control. PCR primers for coding genes and miR-29b precursors used in this study, as well as the size of fragments amplified, are shown in Table S1. Methods for mature miR-29b level detection were as follows: the total RNA was used to detect mature miRNA level using Bulge-Loop miRNA qRT-PCR kit (Guangzhou Ribobio, China). In brief, the real-time miRNA assay has two steps: miRNA RT reaction and real-time PCR detection. miRNA RT primer binds to the 3′end of miRNA molecules and are transcribed with reverse transcriptase. RT product is quantified using real-time PCR with the following thermal cycling profile: 95 °C for 20 sec, followed by 40 cycles of amplification (95 °C for 10 s, 60 °C for 20 s, 70 °C for 10 s). To normalize RNA content, the U6 snRNA was the internal control. The above two kinds of real-time PCR assays were performed using the vii A7 Quantitative PCR System (Applied Biosystems, Carlsbad, CA, USA). The relative expression values of coding genes and mature miRNAs of interest were calculated using the 2^-∆∆Ct^ method [21].

### Western blot analysis

The protein extract (40 µg) prepared from mouse myocardium was separated using 12% SDS-PAGE and transferred onto a polyvinylidene fluoride (PVDF) membrane. The membrane was then blocked for 1 h at room temperature with 5% milk in Tris-buffered saline solution (pH 7.6) containing 0.05% Tween-20 (TBS/T) and incubated with a high affinity anti-Col1a1 antibody (1:1000), anti-Col3a1 antibody (1:1000), anti-α-SMA (1:2000) (Santa Cruz Biotechnology, Santa Cruz, CA, USA), anti-smad3 antibody (1:10 000) and anti-p-smad3 antibody (1:10 000) (Epitomics, Burlingame, CA, USA) overnight at 4 °C. Membranes were then washed extensively with TBS/T and incubated with a horseradish peroxidase (HRP)-conjugated secondary antibody (1:5000) (Santa Cruz Biotechnology, USA) for 1 h at room temperature. Proteins were visualized using the ECL Plus detection system (GE Healthcare, WI, USA). As an internal control, membranes were also immunoblotted with an anti-GAPDH antibody (1: 2000) (Santa Cruz Biotechnology, USA).

### Statistical analysis

The data represent the mean ± standard deviation (SD). In each experiment, all determinations were performed at least in triplicate. Differences between experimental groups were analyzed using Student’s *t*-test. A value of *p*< 0.05 indicated significance.

## Results

### Impaired left ventricular function was rescued by carvedilol treatment

Echocardiography findings are compared in Table 1. The left ventricular (LV) anterior wall end-diastolic thickness (LVAWd) and the systolic LV anterior wall thickness (LVAWs) as assessed by echocardiography were significantly lower in the AMI group (0.36 ± 0.10 and 0.44 ± 0.11, respectively) compared to the sham surgery controls (1.92 ± 0.11 and 2.79 ± 0.28, respectively; *p* < 0.001). However, the carvedilol treatment group had significantly higher LVAWd and LVAWs than did the AMI group (*p* < 0.01, *p* < 0.001, respectively). The LV internal dimension at end-diastole (LVIDd) and the LV internal dimension at end-systole (LVIDs) were also significantly higher in the AMI group (8.08 ± 0.41 and 5.35 ± 0.57, respectively) than in the sham surgery control group (5.94 ± 0.57 and 3.56 ± 0.46, respectively; *p* < 0.001), but the LVIDd and LVIDs were significantly lower in the carvedilol group than in the AMI group (*p* < 0.05, *p* < 0.01, *p* < 0.001, respectively). The EF (%) and FS (%) were significantly lower in the AMI group (55.45±5.04% and 30.19 ± 3.51%, respectively) than in the sham surgery control group (69.91±3.40% and 40.15 ± 2.69%, respectively; *p* < 0.001); medium- and high-dose carvedilol treatment efficiently rescued the AMI-induced reductions of EF (%) and FS (%) (*p* < 0.05, *p* < 0.05, respectively).

**Table 1:** Assessment of the cardiac function by echocardiography (N=8).

Group	Sham	AMI	CAR-L	CAR-M	CAR-H
LVAWd	1.92±0.11	0.36±0.10**^△^**	0.59±0.18^**^	1.54±0.08^***^	1.48±0.16^***^
LVAWs	2.79±0.28	0.44±0.11**^△^**	0.68±0.19^**^	2.25±0.30^***^	1.93±0.20^***^
LVIDd	5.94±0.57	8.08±0.41**^△^**	7.02±0.56^***^	6.69±0.51^***^	7.20±0.41^***^
LVIDs	3.56±0.46	5.35±0.57**^△^**	4.67±0.32^*^	4.34±0.55^**^	4.75±0.55^*^
LVPWd	1.70±0.12	1.94±0.08**^△^**	1.97±0.18	1.84±0.12	1.86±0.12
LVPWs	2.40±0.25	3.08±0.22**^△^**	3.13±0.35	2.92±0.11	3.05±0.38
EF (%)	69.91±3.40	55.45±5.04**^△^**	60.98±7.13	65.60±5.17^*^	64.87±6.26^*^
FS (%)	40.15±2.69	30.19±3.51**^△^**	33.85±5.20	37.03±4.15^*^	36.80±4.78^*^

Data represent the mean±SD, **^Δ^** *p* < 0.001 vs. Sham group, ^*^ *p* < 0.05, ^**^ *p* < 0.01, ^***^ *p* < 0.001 vs. AMI group. N=8.

### ECM-related genes and miR-29b expression in AMI-induced fibrotic myocardium treated with carvedilol

**Figure 1:**
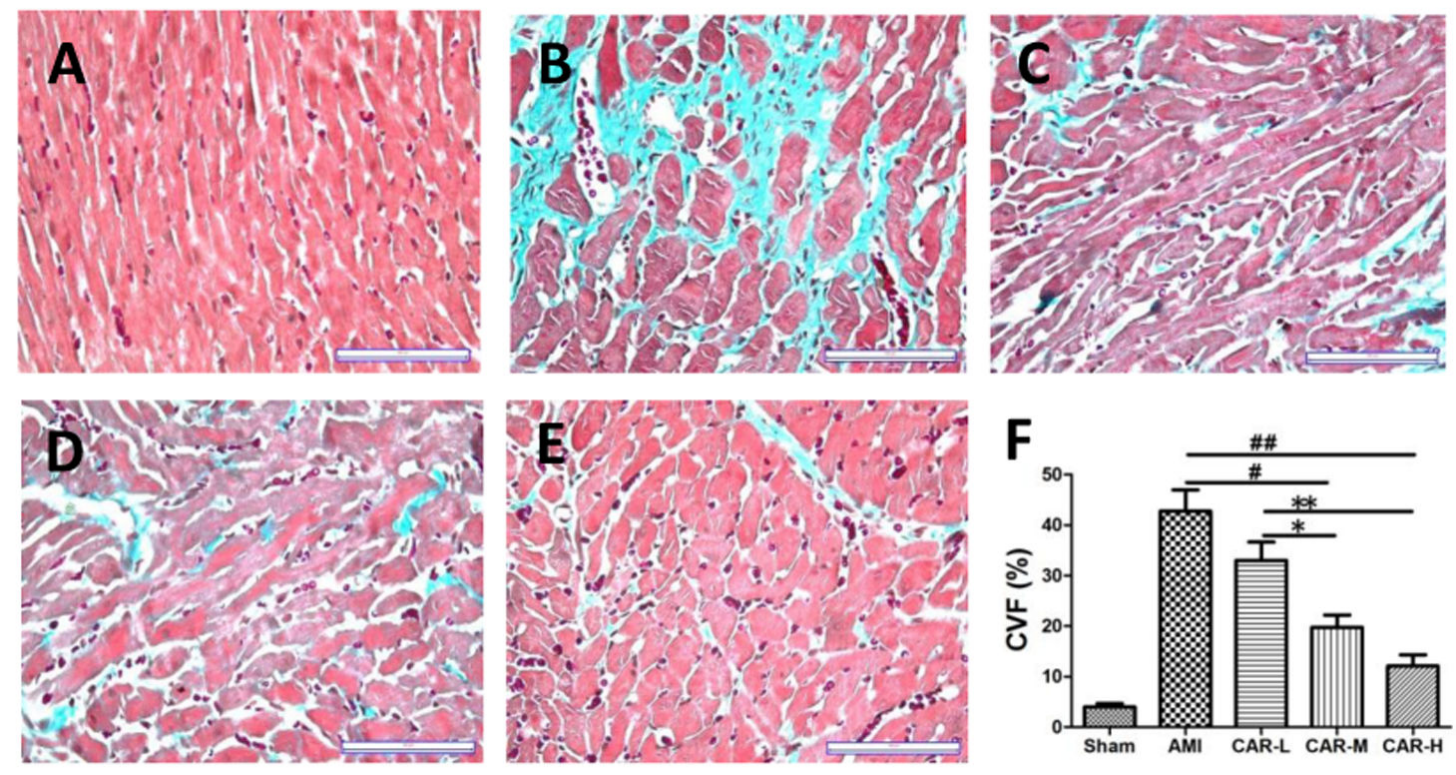
Masson’s trichrome staining of rat heart sections shows scar formation 4weeks after MI and CAR treatment. A-E, the representative views of the sham surgery control, AMI, CAR-L, CAR-M and CAR-H groups, respectively (Scale bar: 100 µm). F. The collagen volume fraction in the border zone of the infarcted myocardial region was significantly lower in the CAR-M and CAR-H groups. ^#^ *p* < 0.01, ^# #^ *p* < 0.001 vs. AMI group, ^*^ *p* < 0.05, ^**^ *p* < 0.01 vs. CAR-L group, N = 4–5.

**Figure 2:**
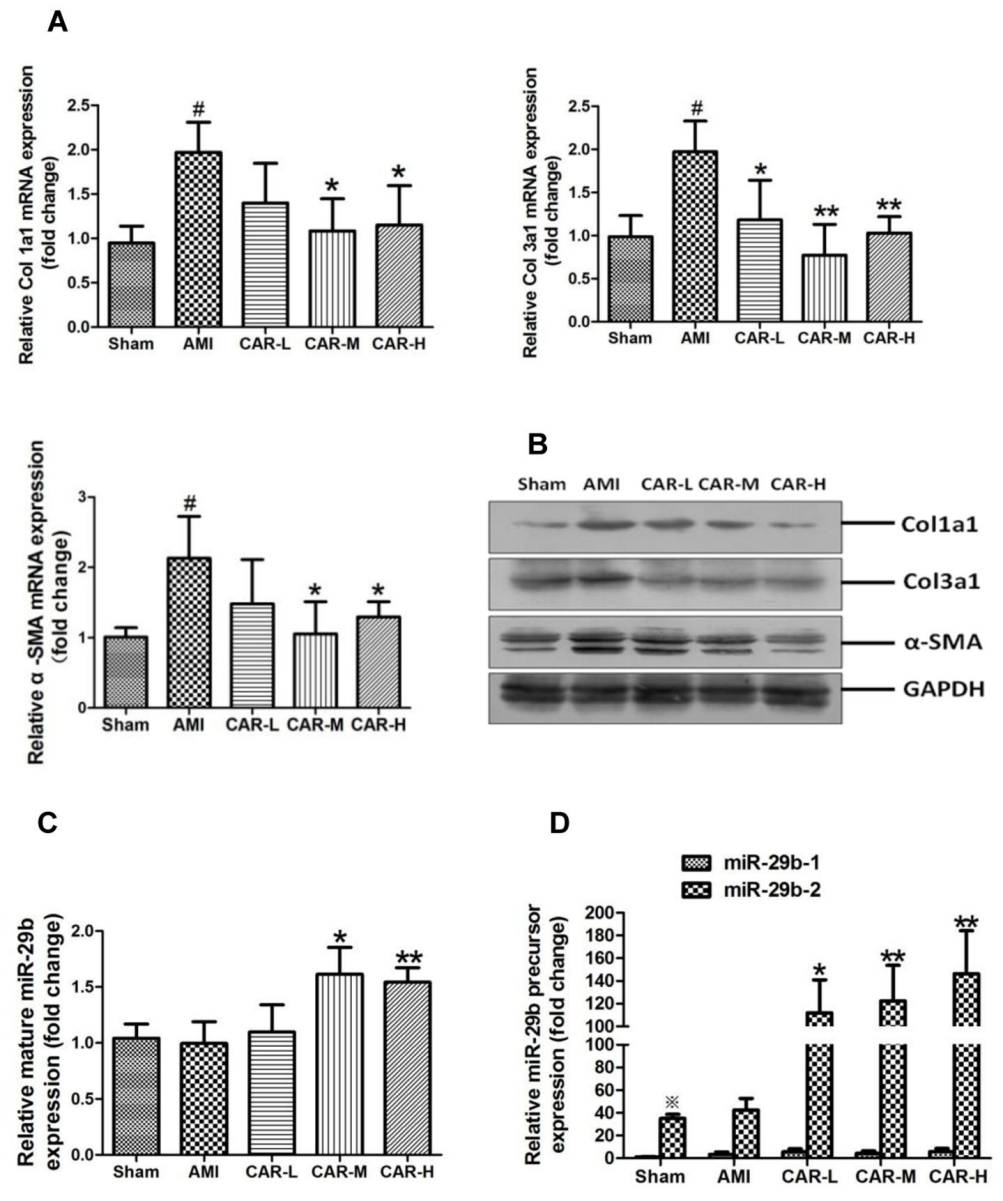
ECM-related Col1a1, Col3a1, and α-SMA expression and miR-29b expression in the border zone of the infarcted region. A. Col1a1, Col3a1, and α-SMA mRNA expression by quantitative real-time PCR assay. ^#^ *p* < 0.01 vs. sham surgery control group; *p < 0.05, **p < 0.01 vs. AMI group, N = 6–8. B. Col1a1, Col3a1, and α-SMA protein expression by Western-blot assay. C. Mature miR-29b expression by quantitative real-time PCR assay. ^*^ *p* < 0.05, ^**^ *p* < 0.01 vs. AMI group, N = 6–8. D. miR-29b-1 and miR-29b-2 precursor expression by quantitative real-time PCR assay. ^※^ *p* < 0.001 vs. miR-29b-1 precursor, *p < 0.05 vs. AMI group, **p < 0.01 vs. AMI group.

Consistent with the echocardiography data, Masson’s trichrome staining showed that the collagen volume fraction (CVF) in the AMI border zone was dramatically lower in the CAR-M and CAR-H AMI groups than in the AMI and CAR-L group (*p* < 0.01 and *p* < 0.001, *p* < 0.05 and *p* < 0.01, respectively) (Figure 1). Quantitative real-time PCR showed that Col1a1, Col3a1, and α-SMA mRNA were significantly decreased in the AMI border zone in the CAR-M and CAR-H groups (*p* < 0.05 and *p* < 0.01, respectively) (Figure 2A). Western-blot results showed that Col1a1, Col3a1, and α-SMA protein expression was also significantly lower in the AMI border zone in the CAR-M and CAR-H groups (Figure 2B, S1).

The quantity of mature miR-29b in the AMI border zone was significantly higher in the CAR-M and CAR-H groups (*p* < 0.05 and *p* < 0.01, respectively) than in the untreated AMI group (Figure 2C). In the sham surgery control group, the expression level of the mir-29b-2 precursor was much higher than that of the mir-29b-1 precursor (*p* < 0.001) (Figure 2D). Only the expression of the miR-29b-2 precursor was significantly higher in all 3 carvedilol-treated AMI groups (*p* < 0.05 and *p* < 0.01, respectively) (Figure 2D).

### ECM-related genes and miR-29b expression in carvedilol-treated rat cardiac fibroblasts *in vitro*

**Figure 3:**
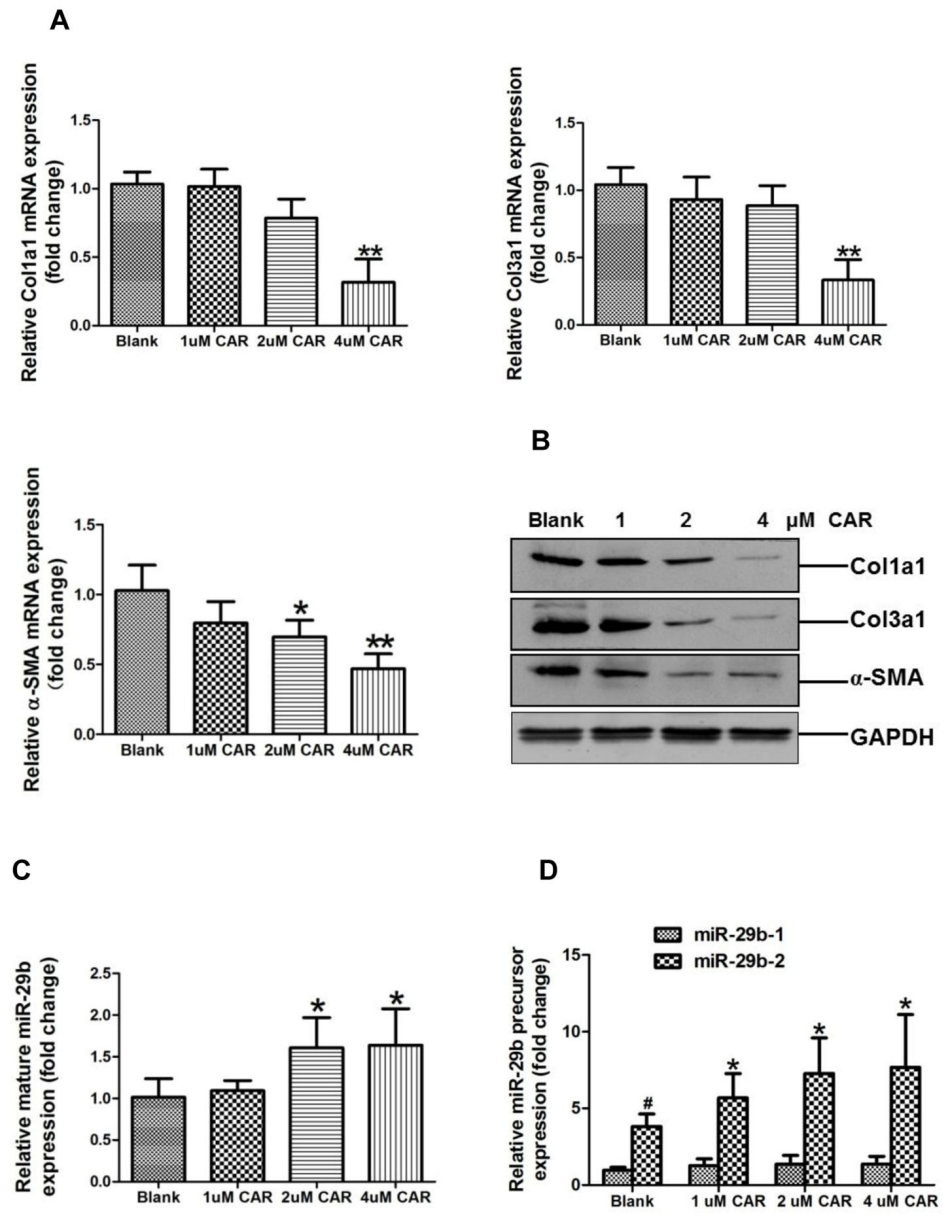
ECM-related Col1a1, Col3a1, and α-SMA expression and miR-29b expression in carvedilol-treated rat cardiac fibroblasts. A. Col1a1, Col3a1, and α-SMA mRNA expression by quantitative real-time PCR assay. *p < 0.05, **p < 0.01 vs. control group, N = 4. B. Col1a1, Col3a1, and α-SMA protein expression by Western-blot assay. C. Mature miR-29b expression by quantitative real-time PCR assay. *p < 0.05 vs. control group, N = 4. D. miR-29b-1 and miR-29b--2 precursor expression by quantitative real-time PCR assay. ^#^ *p* < 0.01 vs. miR-29b-1 in control group; *p < 0.05 vs. control group, N = 4.

A dose-course study of the effect of carvedilol on Col1a1, Col3a1, and α-SMA expression was demonstrated by quantitative real-time PCR and Western-blotting assay, respectively (Figure 3A, Figure 3B, S2). These data demonstrated that 4µM carvedilol inhibited Col1a1, Col3a1, and α-SMA expression at both the mRNA and protein level. Compared with the control group, mature miR-29b was significantly up-regulated in the 2 µM and 4 µM carvedilol-treated rat cardiac fibroblasts (*p* < 0.05 and *p* < 0.05, respectively) (Figure 3C). Quantitative real-time PCR showed that the expression of the mir-29b-2 precursor was much higher than that of the mir-29b-1 precursor (*p* < 0.01) (Figure 3D). Expression of the miR-29b-2 precursor, but not the miR-29b-1 precursor, increased significantly in a dose-dependent manner in carvedilol-treated rat cardiac fibroblasts (*p* < 0.05) (Figure 3D).

### Carvedilol inhibited ROS-activated Smad3 signaling involved in ECM related genes and miR-29b expression

**Figure 4:**
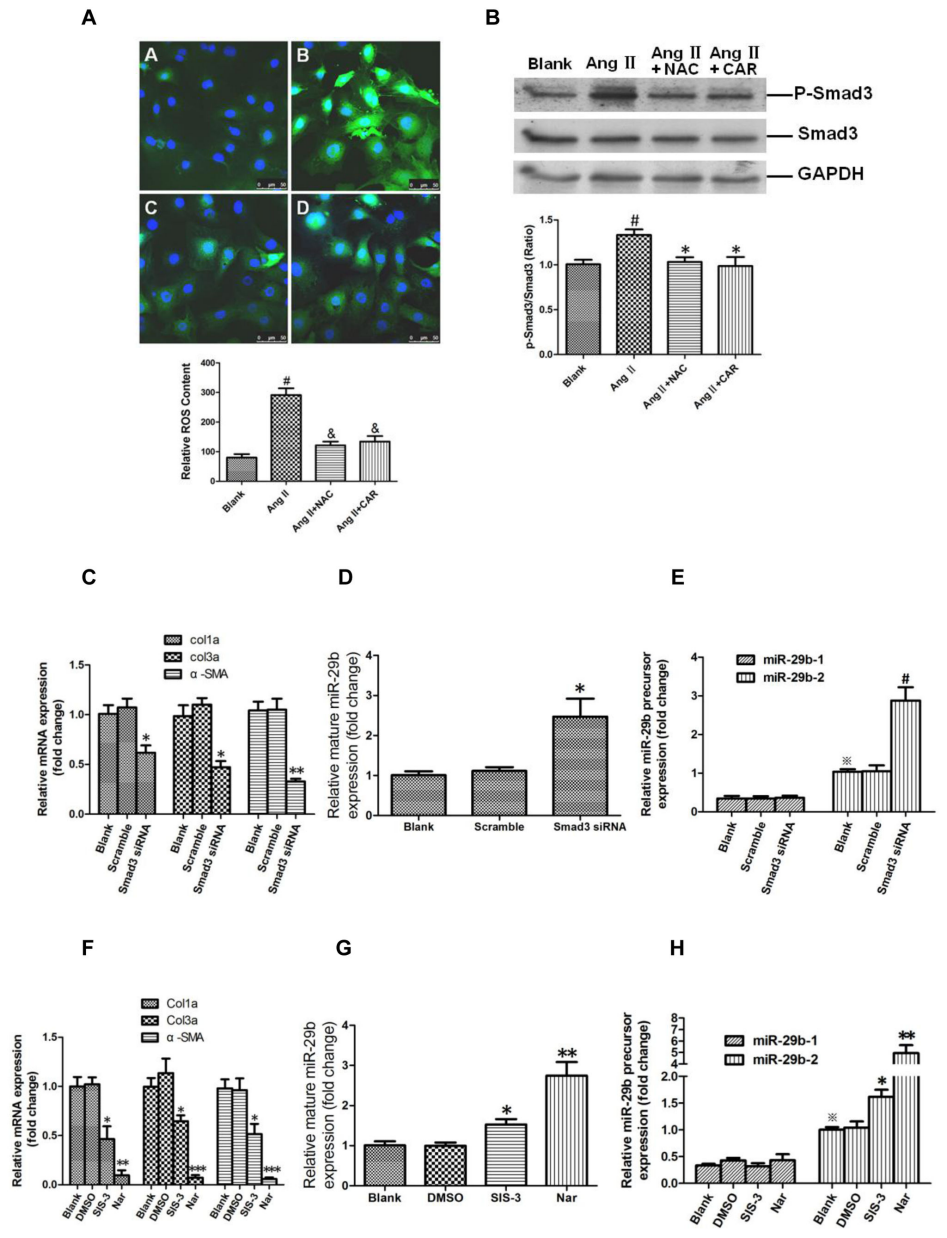
Smad3 signaling pathway, ECM-related genes and miR-29b expression in rat cardiac fibroblasts. A. Measurement of intracellular ROS by DCFH-DA staining (400× magnification). The figures are representative of three independent experiments. ^#^ *p* <0.001 vs. Blank, ^&^ *p* <0.001 vs. Ang II, N=4. B. Inactivation of smad3 in NAC- and carvedilol-treated rat cardiac fibroblasts. ^#^ *p* <0.05 vs. Blank, *p <0.05 vs. Ang II, N=4. C. Downregulation of Col1a1, Col3a1, and α-SMA in smad3 knockdown rat cardiac fibroblasts. *p < 0.05, **p < 0.01 vs. control group, N = 4. D. Upregulation of mature miR-29b in smad3 knockdown rat cardiac fibroblasts. *p < 0.05, **p < 0.01 vs. control group, N = 4. E. Upregulation of miR-29b-2 precursor in smad3 knockdown rat cardiac fibroblasts. ^※^ *p* < 0.01 vs. miR-29b-1 in control group, ^#^ *p* < 0.01 vs. control group, N = 4. F. Downregulation of Col1a1, Col3a1, and α-SMA in SIS-3 or Nar-treated rat cardiac fibroblasts. *p < 0.05, **p < 0.01, ***p < 0.001 vs. Blank group, N = 4. G. Upregulation of mature miR-29b in SIS-3 or Nar-treated rat cardiac fibroblasts. *p < 0.05, **p < 0.01 vs. control group, N = 4. H. Upregulation of miR-29b-2 precursor in SIS-3 or Nar-treated rat cardiac fibroblasts. ^※^ *p* < 0.01 vs. miR-29b-1 in blank control group, *p < 0.05, **p < 0.01 vs. miR-29b-2 in blank control group, N = 4.

By DCFH-DA staining, Ang ii(10^−5^ M) increased intracellular reactive oxygen species (ROS) generation in rat cardiac fibroblasts, while treatment of 10 mM N-acetyl-L-cysteine (NAC) (ROS scavenger) and 4 µM carvedilol reduced ROS production in cardiac fibroblasts induced by Ang ii(*p* < 0.001, respectively, Figure 4A). Western-blot results demonstrated that the Smad3 pathway was activated in Ang ii-treated rat cardiac fibroblasts, NAC or carvedilol treatment decreased p-Smad3 expression (*p* < 0.05, respectively, Figure 4B). Quantitative real-time PCR showed that Col1a1, Col3a1, and α-SMA mRNA expression was significantly lower in Smad3 siRNA-modified rat cardiac fibroblasts (*p* < 0.05, and *p* < 0.01, respectively, Figure 4C). The quantity of mature mir-29b was significantly higher in Smad3 siRNA-modified rat cardiac fibroblasts (*p*<0.05) (Figure 4D). Consistent with this result, the quantity of miR-29b-2 precursor, but not miR-29b-1 precursor, was also dramatically higher in Smad3 siRNA-modified rat cardiac fibroblasts (*p* < 0.01) (Figure 4E). Additionally, Col1a1, Col3a1, and α-SMA mRNA expression was significantly lower in SIS-3 or Naringenin-treated rat cardiac fibroblasts (*p* < 0.05, *p* < 0.01, and *p* < 0.001, respectively) (Figure 4F). The quantity of mature mir-29b and miR-29b-2 precursors, but not miR-29b-1 precursor, increased significantly in SIS-3 or/naringenin-treated rat cardiac fibroblasts (*p* < 0.05 and *p* < 0.01, respectively) (Figure 4G,4H).

### miR-29b modulated expression of ECM-related gene in cardiac fibroblasts

**Figure 5:**
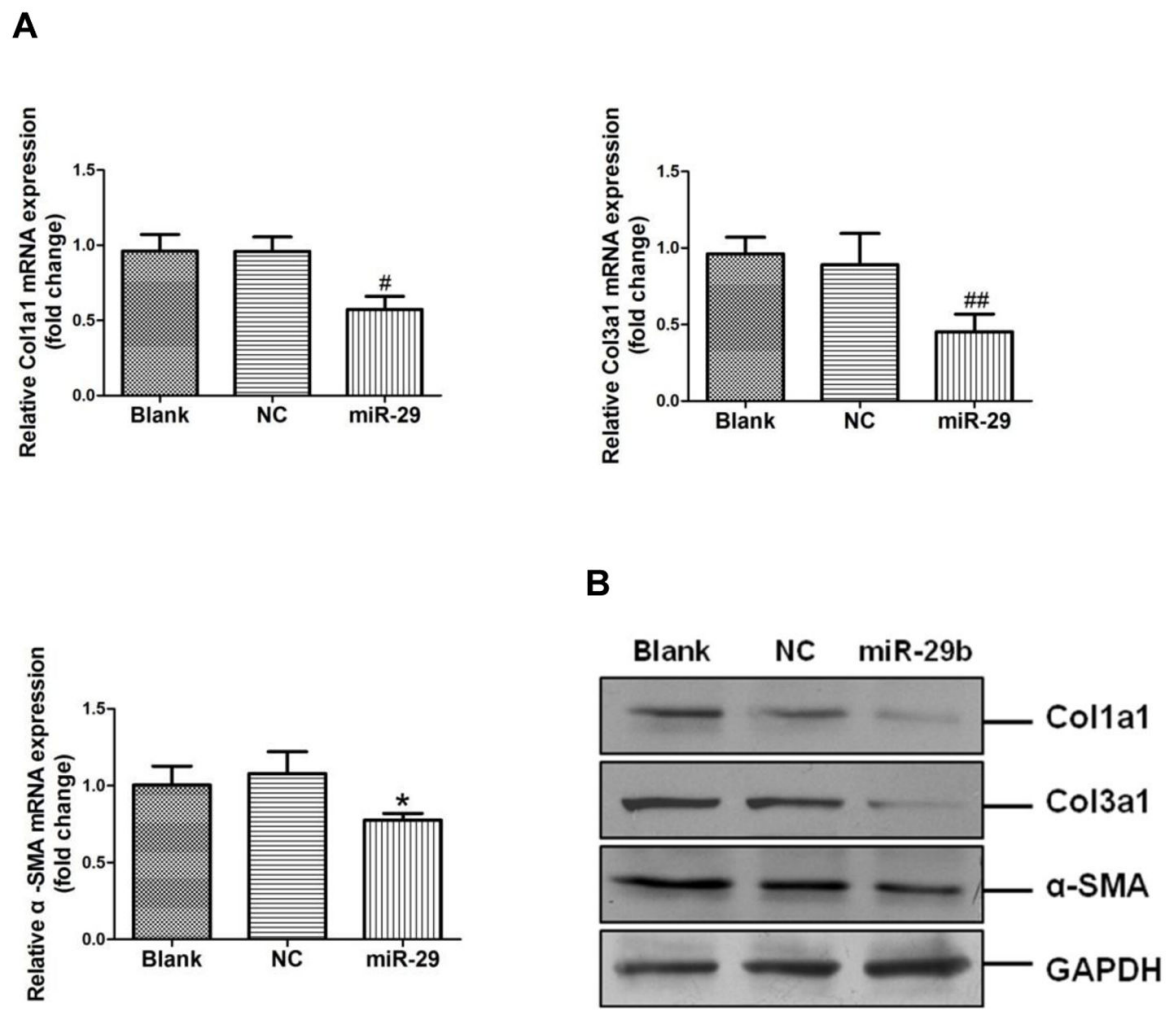
Enforced expression of miR-29b decreased Col1a1, Col3a1, and α-SMA expression. A. Col1a1, Col3a1, and α-SMA mRNA expression by quantitative real-time PCR assay. *p < 0.05, ^#^ *p* < 0.01, ^# #^ *p* < 0.001 vs. control group, N = 3–5. B. Col1a1, Col3a1, and α-SMA protein expression by Western-blot assay. NC, scrambled oligonucleotide.

To study the expression of ECM-related genes after miR-29b mimic treatment, we transfected rat cardiac fibroblasts with the miR-29b mimic or scrambled oligonucleotide. After a 24-h incubation, real-time PCR analysis indicated that the miR-29b mimic inhibited Col1a1, Col3a1, and α-SMA expression at the mRNA level (*p* < 0.05, *p* < 0.01, and *p* < 0.001, respectively) (Figure 5A). Consistent with these results, miR-29b also inhibited Col1a1, Col3a1, and α-SMA protein expression compared to that observed using the scrambled oligonucleotide (*p* < 0.05 and *p* < 0.001, respectively). (Figure 5B, S3.) 

## Discussion

In this study, we demonstrated the anti-fibrotic effect of carvedilol *in vivo* and *in vitro* by inhibiting fibrosis-related genes expression. The fibrosis regulator, miR-29b, was validated to be modulated by carvedilol, contributing to the anti-fibrotic effect of carvedilol. Mechanism study revealed that carvedilol inhibited ROS-induced Smad3 activation, and Smad3 signal negatively modulated miR-29b expression, miR-29b could efficiently inhibit fibrosis-related genes expression in cardiac fibroblasts.

LV remodeling after AMI contributes to heart failure. β-blockers are first-line therapy for AMI and heart failure. Initiation of β-blockers within the first 24 h of the ischemic episode has been recommended in clinical practice guidelines [22]. Carvedilol is a β-blocker that also has α-blocking effects, reducing the mortality rate of patients with moderate to severe chronic cardiac failure by 35% to 65% and lowering re-hospitalization rates [23,24]. The underlying mechanism whereby carvedilol attenuates AMI-induced cardiac remodeling seems to be multifactorial. Carvedilol has antioxidant activity [25], attenuates inflammatory mediators, and activates NF-kB [26]. Carvedilol treatment has been shown to delay oxidative-stress–induced apoptosis by improving Ca^2+^ handling [27] and increasing the expression of anti-apoptosis proteins [28].

Beneficial effects of carvedilol on left ventricular remodeling have been observed in patients with left ventricular dysfunction who had an acute myocardial infarction [29] and chronic heart failure [30]. In animal models of myocardial infarction-induced heart failure, carvedilol treatment was observed to protect against myocardial fibrosis and decrease myocardial collagen in the non-infarcted myocardium [3,4]. Mechanistic studies have demonstrated that carvedilol can inhibit PDGF-induced signal transduction in human cardiac fibroblasts and is thus able to exert an anti-proliferative effect on these cells [31].

Consistent with previous reports [32], the echocardiography results of the present study show that carvedilol treatment can rescue AMI-induced cardiac changes in LVAWd, LVAWs, LVIDd, and LVIDs. In addition, medium- and high-dose carvedilol treatment significantly increased cardiac EF% and FS% compared to those in the untreated AMI group (Table 1). Masson’s staining demonstrated that the collagen volume fraction (CVF) was significantly lower in the border zone of the infarcted myocardium region in the medium- and high-dose carvedilol treatment groups compared with that of the untreated AMI group (Figure 1).

Quantitative real-time PCR and Western-blot assay revealed that Col1a1, Col3a1, and α-SMA expression were significantly lower in the border zone of the infarcted region in the medium- and high-dose carvedilol treatment groups compared with those in the untreated AMI group (Figure 2 A, 2B,S1). This result is similar to that of a previous report that found carvedilol inhibited Col1a1 and Col3a1 mRNA expression in fibrotic kidney [33]. To investigate the anti-fibrotic effect of carvedilol, SD rat cardiac fibroblasts were cultured and treated with carvedilol *in vitro*. Quantitative real-time PCR and Western-blot results revealed that carvedilol inhibited Col1a1, Col3a1, and α-SMA expression in a dose-dependent manner (Figure 3A, 3B,S2).

Activation of the renin-angiotensin system (RAS) has been implicated in the pathogenesis of acute myocardial infarction (AMI) [34]. Angiotensin II (Ang II) played a pivotal role in cardiac fibrosis through increasing the intracellular generation of ROS and stimulating the collagen production in cardiac fibroblasts [35], and ROS could activate TGF-β/smad3 pathway leading to fibrosis [36]. In the present study, we confirmed that Ang II increased ROS generation and Smad3 activation in cardiac fibroblasts (Figure 4A, 4B), however, NAC and cardvedilol could consistently abrogate the effect of Ang II on ROS generation and Smad3 activation (Figure 4A, 4B). Therefore, carvedilol, possessing ROS scavenging and ROS suppressive effects, could inhibit ROS-activated Smad3 signaling involved in cardiac fibrosis.

We further demonstrated that Smad3 siRNA and two Smad3 inhibitors, SIS-3 and Nar, inhibited Col1a1, Col3a1, and α-SMA mRNA expression in rat cardiac fibroblasts (Figure 4C, 4F). These results revealed that inactivation of the Smad3 pathway mediates the anti-fibrotic effect of carvedilol in AMI-induced cardiac fibrosis.

miRNAs have been shown to play roles in cardiac remodeling after AMI [37]. miR-29b was shown to regulate myocardial fibrosis [14,16], but whether carvedilol could modulate miR-29b expression was unknown. In this study, we found that miR-29b was upregulated in the border zone of infarcted myocardium in the rat AMI model treated with medium- and high-dose carvedilol (Fig. 2C). miR-29b was also upregulated by carvedilol in rat cardiac fibroblasts *in vitro* (Fig. 3C). Mature rat miR-29b can be derived from miR-29b-1 and miR-29b-2 precursors, which are transcribed from two different loci in the rat genome (www.mirbase.com). We also confirmed that miR-29b-2 precursor expression was much higher than miR-29b-1 in myocardium (Fig. 2D) and cardiac fibroblasts (Fig. 3D); miR-29b-2, but not miR-29b-1, could be modulated by carvedilol *in vivo* (Fig. 2D) and *in vitro* (Fig. 3D). Using Smad3 siRNA and 2 Smad3 inhibitors, SIS-3 and Nar, we demonstrated that Smad3 signaling negatively regulates miR-29b expression derived from only from the miR-29b-2 precursor (Figure 4E, 4H). This result was consistent with previous reports that Smad3 signaling promotes renal fibrosis by inhibiting miR-29 [15,38].

Previous studies indicated that miR-29b can regulate the expression of ECM-related genes, including Col1a1, Col1a2, and Col3a1 [16,38]. Our results confirm that miR-29b inhibits Col1a1,Col3a1, and α-SMA expression at both the mRNA and protein level (Figure 5A, 5B, S3).

**Figure 6:**
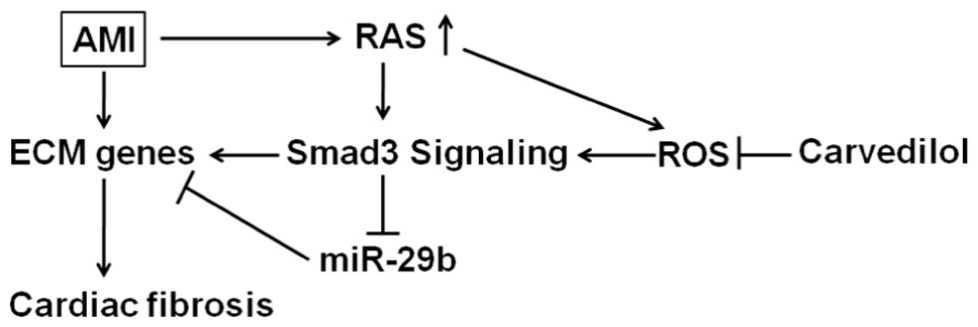
Schematic diagram of the mechanism whereby carvedilol attenuates AMI-induced myocardial fibrosis. Inactivation of ROS-induced smad3 signaling by carvedilol results in suppression of ECM genes, including Col1a1, Col3a1, and α-SMA. Additionally, de-suppression of miR-29b by smad3 inactivation contributes to ECM-related gene expression.

The anti-fibrotic effect of carvedilol contributed to the amelioration of AMI-induced cardiac remodeling and impaired cardiac function in rats. We showed that carvedilol specifically inhibits expression of the ECM-related proteins Col1a1, Col3a1, and α-SMA and promotes miR-29b expression in infarcted myocardium and cardiac fibroblasts *in vitro*. Taken together, these data demonstrate that inactivation of ROS-induced Smad3 signaling and miR-29b upregulation mediate the anti-fibrotic effect of carvedilol in MI-induced cardiac fibrosis (Figure 6).

## Supporting Information

Table S1 **Primers used in real-time qRT-PCR.**
(DOC)

Figure S1 **Col1a1, Col3a1, and α-SMA protein expression in the border zone of the infarcted region by Western-blot assay.** A. Significant inhibition of Col1a1 protein by carvedilol treatment. ^※^ *p* < 0.001 vs. sham surgery control group, ^*^ *p* < 0.05, ^**^ *p* < 0.01, ^***^ *p* < 0.001 vs. AMI group, N = 6–8. B. Significant inhibition of Col3a1 protein by carvedilol treatment. ^※^ *p* < 0.01 vs. sham surgery control group, ^*^ *p* < 0.01, ^**^ *p* < 0.001 vs. AMI group, N = 6–8. C. Significant inhibition of α-SMA protein by medium- and high-dose carvedilol treatment. ^※^ *p* < 0.001 vs. sham surgery control group, ^#^ *p* < 0.01 vs. AMI group, N = 6–8.
(DOC)

Figure S2 **Significant inhibition of Col1a1, Col3a1, and α-SMA protein expression in rat cardiac fibroblasts treated by 2 or 4 µM carvedilol.** ^#^ *p* < 0.01, ^# #^ *p* < 0.001 vs. control group, N = 4.
(DOC)

Figure S3 **Col1a1, Col3a1, and α-SMA protein expression in miR-29b-modified rat cardiofibroblasts.** ※ *p* < 0.001, ^*^ *p* < 0.05 vs. control group, N = 4.
(DOCX)

## References

[1] Packer M, Bristow MR, Cohn JN, Colucci WS, Fowler MB et al. (1996) The effect of carvedilol on morbidity and mortality in patients with chronic heart failure. U.S Carvedilol Heart Failure Study group N Engl J Med 334: 1349–1355 10.1056/NEJM199605233342101 8614419

[2] Chen-Scarabelli C, Saravolatz L Jr, Murad Y, Shieh WS, Qureshi W et al. (2012) A critical review of the use of carvedilol in ischemic heart disease. Am J Cardiovasc Drugs 12: 391–401. doi:10.2165/11636090-000000000-00000. PubMed: 23061698. 23061698 10.1007/BF03262473

[3] Nanjo S, Yamazaki J, Yoshikawa K, Ishii T, Togane Y (2006) Carvedilol prevents myocardial fibrosis in hamsters. Int Heart J 47: 607–616. doi:10.1536/ihj.47.607. PubMed: 16960415. 16960415 10.1536/ihj.47.607

[4] Wei S, Chow LT, Sanderson JE (2000) Effect of carvedilol in comparison with metoprolol on myocardial infarction collagen postinfarction. J Am Coll Cardiol 36: 276–281. doi:10.1016/S0735-1097(00)00671-9. PubMed: 10898446. 10898446 10.1016/s0735-1097(00)00671-9

[5] Bartel DP (2004) MicroRNAs: genomics, biogenesis, mechanism, and function. Cell 116: 281–297. doi:10.1016/S0092-8674(04)00045-5. PubMed: 14744438. 14744438 10.1016/s0092-8674(04)00045-5

[6] Karp X, Ambros V (2005) Developmental biology: encountering microRNAs in cell fate signaling. Science 310: 1288–1289. doi:10.1126/science.1121566. PubMed: 16311325. 16311325 10.1126/science.1121566

[7] Kloosterman WP, Plasterk RH (2006) The diverse functions of microRNAs in animal development and disease. Dev Cell 11: 441–450. doi:10.1016/j.devcel.2006.09.009. PubMed: 17011485. 17011485 10.1016/j.devcel.2006.09.009

[8] van Rooij E, Sutherland LB, Liu N, Williams AH, McAnally J et al. (2007) A signature pattern of stress-responsive microRNAs that can evoke cardiac hypertrophy and heart failure. Proc Natl Acad Sci U S A 103: 18255–18260. 10.1073/pnas.0608791103 PMC1838739 17108080

[9] Feng Y, Yu X (2011) Cardinal roles of miRNA in cardiac development and disease. Sci China Life Sci 54: 1113–1120. doi:10.1007/s11431-011-4354-8. PubMed: 22227903. 22227903 10.1007/s11427-011-4257-8

[10] Calin GA, Croce CM (2006) MicroRNA signatures in human cancers. Nat Rev Cancer 6: 857–866. doi:10.1038/nrc1997. PubMed: 17060945. 17060945 10.1038/nrc1997

[11] Liang H, Zhang C, Ban T, Liu Y, Mei L et al. (2012) A novel reciprocal loop between microRNA-21 and TGFβRIII is involved in cardiac fibrosis. Int J Biochem Cell Biol 44: 2152–2160. doi:10.1016/j.biocel.2012.08.019. PubMed: 22960625. 22960625 10.1016/j.biocel.2012.08.019

[12] Shan ZX, Lin QX, Fu YH, Deng CY, Zhou ZL et al. (2009) Upregulated expression of miR-1/miR-206 in a rat model of myocardial infarction. Biochem Biophys Res Commun 381: 597–601. doi:10.1016/j.bbrc.2009.02.097. PubMed: 19245789. 19245789 10.1016/j.bbrc.2009.02.097

[13] Shi B, Guo Y, Wang J, Gao W (2010) Altered expression of microRNAs in the myocardium of rats with acute myocardial infarction. BMC Cardiovasc Disord 10: 11. doi:10.1186/1471-2261-10-11. PubMed: 20187981. 20187981 10.1186/1471-2261-10-11 PMC2844352

[14] van Rooij E, Sutherland LB, Thatcher JE, DiMaio JM, Naseem RH et al. (2008) Dysregulation of microRNAs after myocardial infarction reveals a role of miR-29 in cardiac fibrosis. Proc Natl Acad Sci U S A 105: 13027–13032. doi:10.1073/pnas.0805038105. PubMed: 18723672. 18723672 10.1073/pnas.0805038105 PMC2529064

[15] Xiao J, Meng XM, Huang XR, Chung AC, Feng YL et al. (2012) miR-29 inhibits bleomycin-induced pulmonary fibrosis in mice. Mol Ther 20: 1251–1260. doi:10.1038/mt.2012.36. PubMed: 22395530. 22395530 10.1038/mt.2012.36 PMC3369297

[16] Kriegel AJ, Liu Y, Fang Y, Ding X, Liang M (2012) The miR-29 family: genomics, cell biology, and relevance to renal and cardiovascular injury. Physiol Genomics 44: 237–244. doi:10.1152/physiolgenomics.00141.2011. PubMed: 22214600. 22214600 10.1152/physiolgenomics.00141.2011 PMC3289120

[17] Dobaczewski M, Chen W, Frangogiannis NG (2011) Transforming growth factor (TGF)-β signaling in cardiac remodeling. J Mol Cell Cardiol 51: 600–606. doi:10.1016/j.yjmcc.2010.10.033. PubMed: 21059352. 21059352 10.1016/j.yjmcc.2010.10.033 PMC3072437

[18] Fu YH, Lin QX, Li XH, Fei HW, Shan ZX et al. (2009) A novel rat model of chronic heart failure following myocardial infarction. Methods Find Exp Clin Pharmacol 31: 367–373. PubMed: 19798451. 19798451 10.1358/mf.2009.31.6.1393631

[19] Feuerstein GZ, Ruffolo RR Jr (1995) Carvedilol, a novel multiple action antihypertensive agent with antioxidant activity and the potential for myocardial and vascular protection. Eur Heart J 16 Suppl F: 38–42. doi:10.1093/eurheartj/16.1.38. PubMed: 8521883. 10.1093/eurheartj/16.suppl_f.38 8521883

[20] Zmijewski JW, Moellering DR, Le Goffe C, Landar A, Ramachandran A et al. (2005) Oxidized LDL induces mitochondrially associated reactive oxygen/nitrogen species formation in endothelial cells. Am J Physiol Heart Circ Physiol 289: H852–H861. doi:10.1152/ajpheart.00015.2005. PubMed: 15805232. 15805232 10.1152/ajpheart.00015.2005

[21] Pfaffl MW (2001) A new mathematical model for relative quantification in real-time RT-PCR. Nucleic Acids Res 29: e45. doi:10.1093/nar/29.9.e45. PubMed: 11328886. 11328886 10.1093/nar/29.9.e45 PMC55695

[22] Antman EM, Hand M, Armstrong PW, Bates ER, Green LA et al. (2008) 2007 focused update of the ACC/AHA 2004 guidelines for the management of patients with ST-elevation myocardial infarction: a report of the American College of Cardiology/American Heart Association Task Force on Practice Guidelines: developed in collaboration with the Canadian Cardiovascular Society endorsed by the American Academy of Family Physicians: 2007 Writing Group to Review New Evidence and Update the ACC/AHA 2004 Guidelines for the Management of Patients With ST-Elevation Myocardial Infarction, writing on behalf of the 2004 Writing Committee. Circulation 117: 296–329. doi:10.1161/CIRCULATIONAHA.107.188209. PubMed: 18071078. 18071078 10.1161/CIRCULATIONAHA.107.188209

[23] Colucci WS, Packer M, Bristow MR, Gilbert EM, Cohn JN et al. (1996) Carvedilol inhibits clinical progression in patients with mild symptoms of heart failure. US Carvedilol Heart Failure Study Group. Circulation 94: 2800–2806. doi:10.1161/01.CIR.94.11.2800. PubMed: 8941105. 8941105 10.1161/01.cir.94.11.2800

[24] Packer M, Fowler MB, Roecker EB, Coats AJ, Katus HA et al. (2002) Carvedilol Prospective Randomized Cumulative Survival (COPERNICUS) Study Group. Effect of carvedilol on the morbidity of patients with severe chronic heart failure: results of the carvedilol prospective randomized cumulative survival (COPERNICUS) study. Circulation 106: 2194–2199. doi:10.1161/01.CIR.0000035653.72855.BF. PubMed: 12390947. 12390947 10.1161/01.cir.0000035653.72855.bf

[25] Yue TL, Cheng HY, Lysko PG, McKenna PJ, Feuerstein R et al. (1992) Carvedilol, a new vasodilator and beta adrenoceptor antagonist, is an antioxidant and free radical scavenger. J Pharmacol Exp Ther 263: 92–98. PubMed: 1357162. 1357162

[26] Zhuang XF, Yin CQ, Wang HY, Sun NL (2009) Distinctive effects of carvedilol in the non-infarct zone: Remodeling of the ligated rat heart linked to oxidative stress. J Int Med Res 37: 1354–1364. doi:10.1177/147323000903700510. PubMed: 19930840. 19930840 10.1177/147323000903700510

[27] Wang R, Miura T, Harada N, Kametani R, Shibuya M et al. (2006) Pleiotropic effects of the beta-adrenoceptor blocker carvedilol on calcium regulation during oxidative stress-induced apoptosis in cardiomyocytes. J Pharmacol Exp Ther 318: 45–52. doi:10.1124/jpet.105.099903. PubMed: 16611853. 16611853 10.1124/jpet.105.099903

[28] Zhang JL, Lu JK, Chen D, Cai Q, Li TX et al. (2009) Myocardial autophagy variation during acute myocardial infarction in rats: The effects of carvedilol. Chin Med J (Engl) 122: 2372–2379. PubMed: 20079142. 20079142

[29] Senior R, Basu S, Kinsey C, Schaeffer S, Lahiri A (1999) Carvedilol prevents remodeling in patients with left ventricular dysfunction after acute myocardial infarction. Am Heart J 137: 646–652. doi:10.1016/S0002-8703(99)70217-6. PubMed: 10097224. 10097224 10.1016/s0002-8703(99)70217-6

[30] Lowes BD, Gill EA, Abraham WT, Larrain JR, Robertson AD et al. (1999) Effects of carvedilol on left ventricular mass, chamber geometry, and mitral regurgitation in chronic heart failure. Am J Cardiol 83: 1201–1205. doi:10.1016/S0002-9149(99)00059-4. PubMed: 10215284. 10215284 10.1016/s0002-9149(99)00059-4

[31] Lotze U, Heinke S, Fritzenwanger M, Krack A, Müller S et al. (2002) Carvedilol inhibits platelet-derived growth factor-induced signal transduction in human cardiac fibroblasts. J Cardiovasc Pharmacol 39: 576–589. doi:10.1097/00005344-200204000-00014. PubMed: 11904532. 11904532 10.1097/00005344-200204000-00014

[32] Li B, Liao YH, Cheng X, Ge H, Guo H et al. (2006) Effects of carvedilol on cardiac cytokines expression and remodeling in rat with acute myocardial infarction. Int J Cardiol 111: 247–255. doi:10.1016/j.ijcard.2005.08.065. PubMed: 16310260. 16310260 10.1016/j.ijcard.2005.08.065

[33] Wong VY, Laping NJ, Nelson AH, Contino LC, Olson BA et al. (2001) Renoprotective effects of carvedilol in hypertensive-stroke prone rats may involve inhibition of TGF beta expression. Br J Pharmacol 134: 977–984. doi:10.1038/sj.bjp.0704329. PubMed: 11682445. 11682445 10.1038/sj.bjp.0704329 PMC1573025

[34] Vaughan DE, Rouleau JL, Pfeffer MA (1995) Role of the fibrinolytic system in preventing myocardial infarction. Eur Heart J 16 Suppl K: 31–36. doi:10.1093/eurheartj/16.suppl_I.31. PubMed: 8869133. 10.1093/eurheartj/16.suppl_k.31 8869133

[35] Lijnen PJ, van Pelt JF, Fagard RH (2012) Stimulation of reactive oxygen species and collagen synthesis by angiotensin II in cardiac fibroblasts. Cardiovasc Ther 30: e1–e8. doi:10.1111/j.1755-5922.2010.00231.x. PubMed: 20626399. 20626399 10.1111/j.1755-5922.2010.00205.x

[36] Gao Fei, Vuokko L, Kinnula, Myllärniemi M, Oury TD (2008) Extracellular Superoxide Dismutase in Pulmonary Fibrosis. Antioxid Redox Signal 10: 343–354. doi:10.1089/ars.2007.1908. PubMed: 17999630. 17999630 10.1089/ars.2007.1908 PMC2290736

[37] Zhu H, Fan GC (2012) Role of microRNAs in the reperfused myocardium towards post-infarct remodelling. Cardiovasc Res 94: 284–292. doi:10.1093/cvr/cvr291. PubMed: 22038740. 22038740 10.1093/cvr/cvr291 PMC3331611

[38] Qin W, Chung AC, Huang XR, Meng XM, Hui DS et al. (2011) TGF-β/Smad3 signaling promotes renal fibrosis by inhibiting miR-29. J Am Soc Nephrol 22: 1462–1474. doi:10.1681/ASN.2010121308. PubMed: 21784902. 21784902 10.1681/ASN.2010121308 PMC3148701

