# Supplementary material for: Smad3 Inactivation and MiR-29b Upregulation Mediate the Effect of Carvedilol on Attenuating the Acute Myocardium Infarction-Induced Myocardial Fibrosis in Rat
Source: PLoS One. 2013 Sep 25;8(9):e75557. doi: 10.1371/journal.pone.0075557 (PMC3783413; doi:10.1371/journal.pone.0075557)
Supplement: Table S1 — Primers used in real-time qRT-PCR. (DOC) [file pone.0075557.s001.doc]

**Supplementary**

**Table S**1 Primers used in real-time qRT-PCR

| Gene | Sequence (5′- 3′) | Product size (bp） |
| --- | --- | --- |
| Col1a1 | F, GGTCAGACCTGTGTGTTCCC  R, GGTCCATGTAGGCTACGCTG | 250 |
| Col3a1 | F, CAATGTAAAGAAGTCTCTGAAG  R, CAAACAGGGCCAATGTCCAC | 240 |
| α-SMA | F, CTGTGCTATGTCGCTCTGGA  R, ATAGGTGGTTTCGTGGATGC | 192 |
| miR-29b-1 pre | F, CAGCTCTGCCACAGTGAATG  R, GTTCTCGTCCTCTTCCAACG | 202 |
| miR-29b-2 pre | F, CAGGGAAACAACCTCTGCAT  R, ACCATGTGAAACCAGCTTCC | 190 |
| GAPDH | F, CAAGAAGGTGGTGAAGCAGG  R, CCACCCTGTTGCTGTAGCC | 200 |
