# Supplementary material for: Smad3 Inactivation and MiR-29b Upregulation Mediate the Effect of Carvedilol on Attenuating the Acute Myocardium Infarction-Induced Myocardial Fibrosis in Rat
Source: PLoS One. 2013 Sep 25;8(9):e75557. doi: 10.1371/journal.pone.0075557 (PMC3783413; doi:10.1371/journal.pone.0075557)
Supplement: Figure S1 — Col1a1, Col3a1, and α-SMA protein expression in the border zone of the infarcted region by Western-blot assay. A. Significant inhibition of Col1a1 protein by carvedilol treatment. ※ p < 0.001 vs. sham surgery control group, * p < 0.05, ** p < 0.01, *** p < 0.001 vs. AMI group, N = 6–8. B. Significant inhibition of Col3a1 protein by carvedilol treatment. ※ p < 0.01 vs. sham surgery control group, * p < 0.01, ** p < 0.001 vs. AMI group, N = 6–8. C. Significant inhibition of α-SMA protein by medium- and high-dose carvedilol treatment. ※ p < 0.001 vs. sham surgery control group, # p < 0.01 vs. AMI group, N = 6–8. (DOC) [file pone.0075557.s002.doc]

**Supplementary Figure (S1).**

A. B. C.


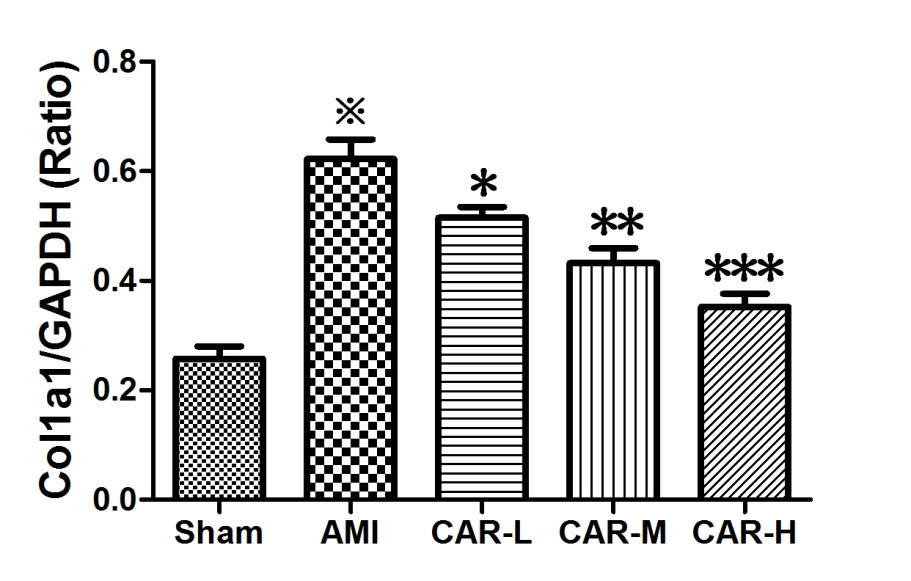

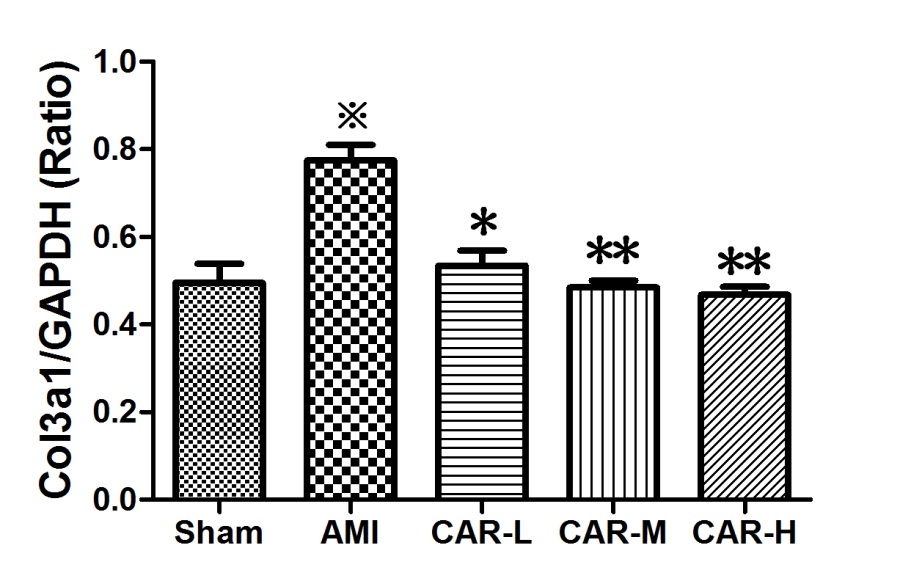

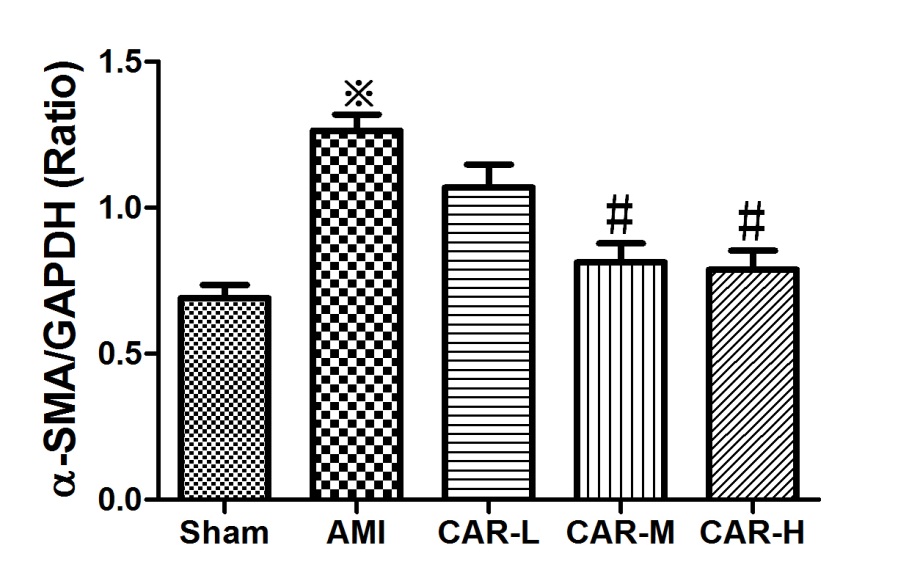


**Figure S1**. Col1a1, Col3a1, and α-SMA protein expression in the border zone of the infarcted region by Western-blot assay. A. Significant inhibition of Col1a1 protein by carvedilol treatment. ^※^*p* < 0.001 vs. sham surgery control group, ^*^*p* < 0.05, ^**^*p* < 0.01, ^***^*p* < 0.001 vs. AMI group, N = 6–8. B. Significant inhibition of Col3a1 protein by carvedilol treatment. ^※^*p* < 0.01 vs. sham surgery control group, ^*^*p* < 0.01, ^**^*p* < 0.001 vs. AMI group, N = 6–8. C. Significant inhibition of α-SMA protein by medium- and high-dose carvedilol treatment. ^※^*p* < 0.001 vs. sham surgery control group, ^#^*p* < 0.01 vs. AMI group, N = 6–8.
