# Supplementary material for: Smad3 Inactivation and MiR-29b Upregulation Mediate the Effect of Carvedilol on Attenuating the Acute Myocardium Infarction-Induced Myocardial Fibrosis in Rat
Source: PLoS One. 2013 Sep 25;8(9):e75557. doi: 10.1371/journal.pone.0075557 (PMC3783413; doi:10.1371/journal.pone.0075557)
Supplement: Figure S2 — Significant inhibition of Col1a1, Col3a1, and α-SMA protein expression in rat cardiac fibroblasts treated by 2 or 4 µM carvedilol. # p < 0.01, # # p < 0.001 vs. control group, N = 4. (DOC) [file pone.0075557.s003.doc]

**Supplementary Figure (S2).**

1. B. C.


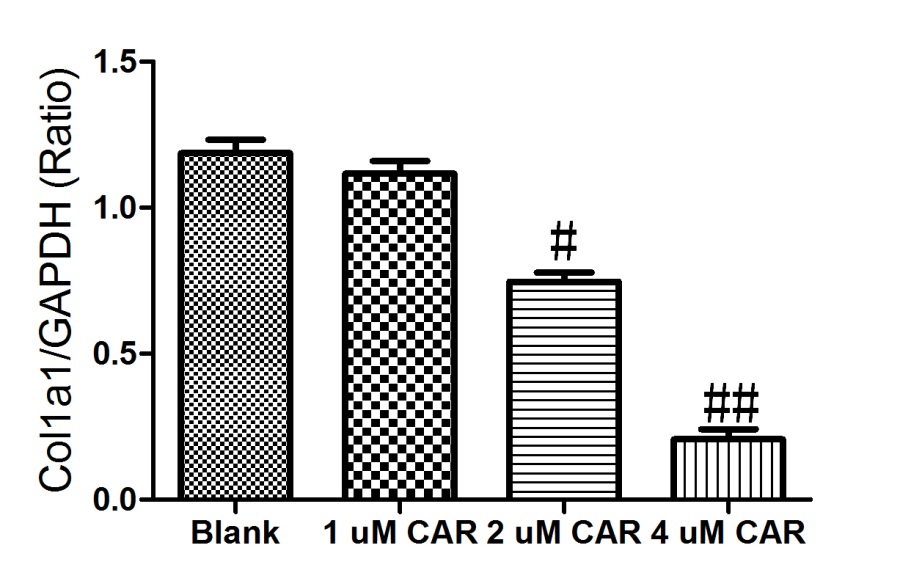

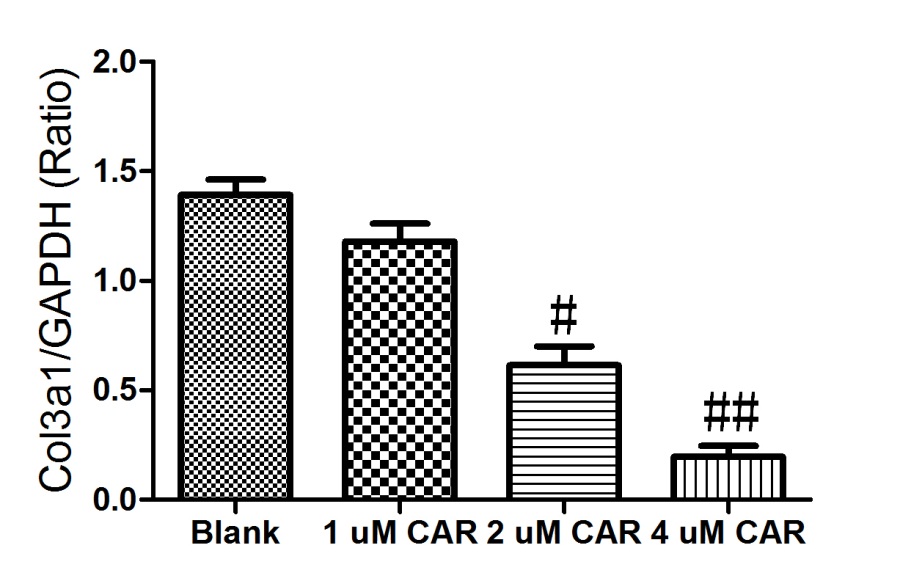

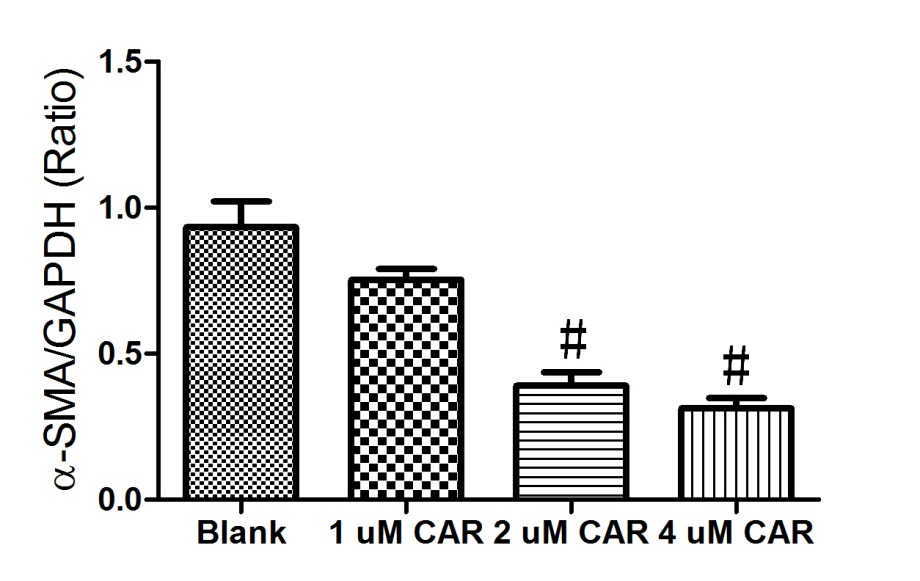


**Figure S2**. Significant inhibition of Col1a1, Col3a1, and α-SMA protein expression in rat cardiac fibroblasts treated by 2 or 4 μM carvedilol. ^#^*p* < 0.01, ^##^*p* < 0.001 vs. control group, N = 4.
