# Supplementary material for: Smad3 Inactivation and MiR-29b Upregulation Mediate the Effect of Carvedilol on Attenuating the Acute Myocardium Infarction-Induced Myocardial Fibrosis in Rat
Source: PLoS One. 2013 Sep 25;8(9):e75557. doi: 10.1371/journal.pone.0075557 (PMC3783413; doi:10.1371/journal.pone.0075557)
Supplement: Figure S3 — Col1a1, Col3a1, and α-SMA protein expression in miR-29b-modified rat cardiofibroblasts. ※ p < 0.001, * p < 0.05 vs. control group, N = 4. (DOCX) [file pone.0075557.s004.docx]

**Supplementary Figure (S3).**

1. B. C.


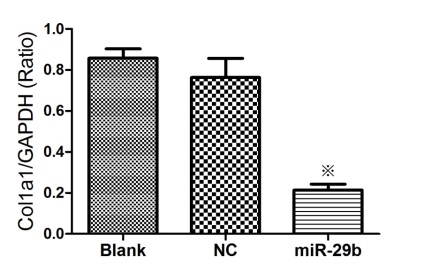

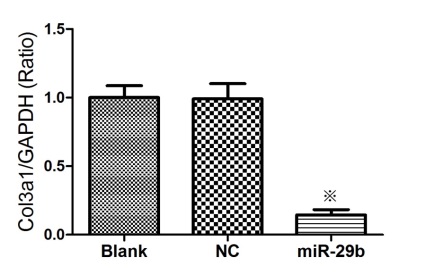

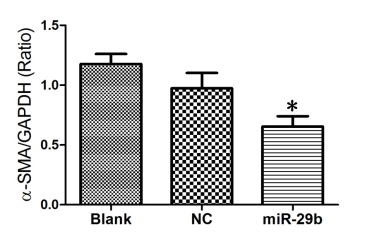


**Figure S3**. Col1a1, Col3a1, and α-SMA protein expression in miR-29b-modified rat cardiofibroblasts. ^※^*p* < 0.001, ^*^*p* < 0.05 vs. control group, N = 4.
